# An Effective Retinal Blood Vessel Segmentation by Using Automatic Random Walks Based on Centerline Extraction

**DOI:** 10.1155/2020/7352129

**Published:** 2020-03-21

**Authors:** Jianqing Gao, Guannan Chen, Wenru Lin

**Affiliations:** ^1^Smart Home Information Collection and Processing on Internet of Things Laboratory of Digital Fujian, Fujian Jiangxia University, Fuzhou 350108, China; ^2^School of Electronic Information Science, Fujian Jiangxia University, Fuzhou 350108, China; ^3^Key Laboratory of OptoElectronic Science and Technology for Medicine of Ministry of Education, Fujian Provincial Key Laboratory for Photonics Technology, Fujian Normal University, Fuzhou 350007, China; ^4^Fujian Provincial Engineering Technology Research Center of Photoelectric Sensing Application, Fujian Normal University, Fuzhou 350117, China; ^5^College of Computer and Control Engineering, Minjiang University, Fuzhou 350121, China; ^6^Fujian Provincial Key Laboratory of Information Processing and Intelligent Control, Minjiang University, Fuzhou 350121, China

## Abstract

The retinal blood vessel analysis has been widely used in the diagnoses of diseases by ophthalmologists. According to the complex morphological characteristics of the blood vessels in normal and abnormal images, an automatic method by using the random walk algorithms based on the centerlines is proposed to segment retinal blood vessels. Hessian-based multiscale vascular enhancement filtering is used to display the vessel structures in maximum intensity projection. Random walk algorithm provides a unique and quality solution, which is robust to weak object boundaries. Seed groups in the random walk segmentation are labeled according to the centerlines, which are extracted by using the divergence of the normalized gradient vector field and the morphological method. Experiments of the proposed method are implemented on the publicly available STARE (the Structured Analysis of the Retina) database. The results are compared to other existing retinal blood vessel segmentation methods with respect to the accuracy, sensitivity, and specificity, and the proposed method is proved to be more sensitive in detecting the retinal blood vessels in both normal and pathological areas.

## 1. Introduction

The network of blood vessels in the fundus is the only part of the human body where the microcirculation can be observed directly [1]. The morphological changes of the retinal blood vessels are closely related to the characterization of certain pathologies [2]. Since automated segmentation of fundus images can provide the basis for automated assessment by doctors, detecting blood vessels automatically from a retinal image is of great research value.

There are a lot of previous works on extracting blood vessels in retinal images, which can be classified into both unsupervised and supervised methods [3–6]. Unsupervised methods mainly include matched filter responses, mathematical morphology-based techniques, model-based locally adaptive thresholding, and vessel tracking. In [2, 7, 8], matched filters are used to enhance the vessel-like objects, the kernel of which is usually the Gaussian-shaped function. In paper [9], a Hessian-based vascular filtering method is used to enhance the vessel structure. Zhang et al. in [10] proposed new filters based on 3D rotating frames in so-called orientation scores, which can handle typical difficult situations such as intersections, central arterial reflux, tight parallelism, and tiny blood vessels. Mathematical morphology is applied in paper [11], which is used to highlight vessels with respect to their morphological properties, such as linearity, connectivity, and width. Neto et al. [12] used a coarse-to-fine approach to blood vessels in fundus images, which combines Gaussian smoothing, a morphological top-hat operator, and vessel contrast enhancement. In model-based locally adaptive thresholding method proposed by papers [13, 14], a set of local information is tested to determine the threshold of the probe region. Vessel tracking presented in [15, 16] uses the profile model, guided by local information, to follow the path which best matches a vessel and segment it incrementally. Abdallah et al. in [17] applied adaptive noise-reducing anisotropic diffusion filter and multiscale line-tracking algorithm to the retinal vessel extraction process. Supervised methods for retinal vessel segmentation use vessel data to train a classifier to identify whether a pixel is vessel or nonvessel, such as support vector machine-based methods and neural network-based methods. A structured output support vector machine is used in [18] to automatically learn the parameters of a trained segmentation model based on a fully connected CRF. A u-net architecture is proposed in [19], which requires very few annotated images. Soomro et al. [20] presented a method using deep conventional neural networks along with hysteresis threshold method for accurate detection of the narrowly low-contrast vessels. Leopold et al. [21] proposed an efficient depth method for automatic segmentation of fundus morphology called PixelBNN, which can be well implemented even in the case of severe information loss. Although supervised methods can provide better results, such methods require training with manually labeled images and may not be available in all cases.

Unfortunately, because of the complex morphological properties of the blood vessels and the impacts of uneven illumination, as well as the appearance of pathological areas in the retinal images, none of the existing methods can achieve the most satisfying performance in all aspects so far. In this paper, an automated segmentation method using random walks based on centerline extraction is proposed, which belongs to the first category. Centerlines are used to assure the location of the vessel, which are extracted by using the combination of the multiscale Hessian-based tubular filters and the divergence value of normalized gradient vector field. And random walks are applied to the segmentation of the fundus images. Most details of the vessels in the low-contrast areas can be detected in healthy and pathological areas.

## 2. Materials and Methods

**Figure 1:**
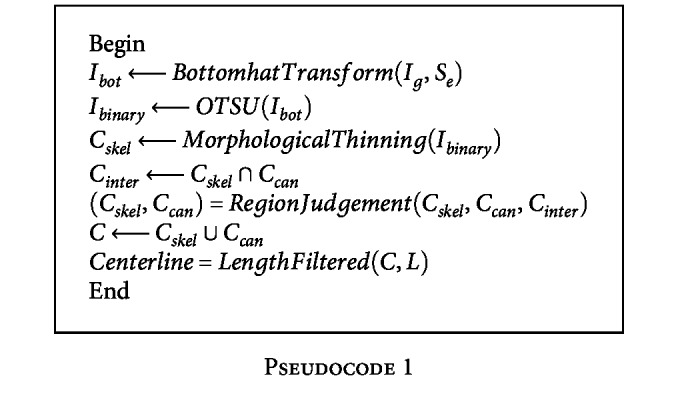
The framework of the proposed method.

The framework of the proposed method mainly includes four steps as shown in Figure 1. Firstly, the original images are converted to grayscale images, and the FOV mask is extracted. Secondly, blood vessel-like objects are enhanced by using the Hessian-based multiscale vessel enhancement filter [22]. Thirdly, centerlines of the vessels are obtained based on the divergence value of the normalized gradient vector field (GVF) and bottom-hat operators with different scales in different orientations. Finally, the seed groups are automatically located, and the random walk algorithm is used to segment the blood vessels.

### 2.1. Image Acquisition and Preparation

Experiments in our work are implemented on images from the publicly available retinal image database, STARE. The digitized slides were captured by a TopCon TRV-50 fundus camera at 35° field of view. Each slide was digitized to produce a 605^×^700 pixel image, 24 bits per pixel (standard RGB) [2, 8]. The results of the proposed method are also compared with several other existing approaches and the hand-labeled vessel networks provided by two experts which are available in the STARE database.

**Figure 2:**
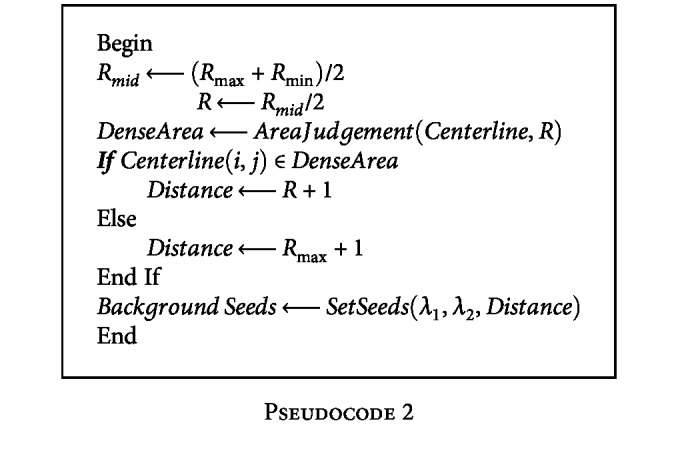
An example of input images and FOV masks: (a) the input image of im0001; (b) the FOV mask of im0001.

The green channel has better performance in the contrast between vessels and the retinal background; therefore, we use the green channel of the RGB retinal image as the input image, denoted as *I*_*g*_. The green channel of im0001 in the STARE database is used as an example, as shown in Figure 2(a). Since the STARE datasets do not provide the FOV-mask images, we use the algorithm proposed by Soares et al. [23] to generate the FOV mask of each retina images, as shown in Figure 2(b).

### 2.2. Vessel Enhancement Filtering

Frangi et al. [22] designed a multiscale vessel enhancement filter based on the eigenvalues of the Hessian matrix. As Hessian matrix can be mathematically decomposed into the isotropic part $\bar{H}$ and the anisotropic part $\overset{\text{\textasciitilde{}}}{H}$, the shape and orientation information of Hessian matrix can be mathematically analyzed as diffusion tensors [24], which is applied in the novel filter proposed in [25] as follows.

Firstly, since a blood vessel usually appears darker than the background in the captured images and, thus, has a concave shape, the green channel of the retinal image can be smoothed by the matched Gaussian filter with standard deviation *σ*, the result of which is denoted as *g*. *σ* also represents different scales in the kernel.

For a certain scale *σ*, the Hessian matrix for a pixel at (*x*, *y*) is given by

(1) $$\begin{array}{c} H\left(x,y\right)=\left[\begin{array}{cc} g_{xx} & g_{xy}\\ g_{yx} & g_{yy} \end{array}\right], \end{array}$$

where *g*_*xx*_, *g*_*yx*_, *g*_*xy*_, and *g*_*yy*_ denote second derivative of *I*_*g*_, respectively.

Secondly, the following combination of the eigenvalues of the Hessian matrix is used to define a vesselness function, which is given by [25]:

(2) $$\begin{array}{c} \nu \left(\sigma \right)=\left\{\begin{array}{c} 0,\;\lambda_{1}>0,\\ \left(1‐e^{-\left(s^{2}/2c^{2}\right)}\right)e^{-\left|\text{FAH}-1\right|},\;\lambda_{1}\leq 0, \end{array}\right. \end{array}$$

where $s=\sqrt{{\lambda_{1}}^{2}+{\lambda_{2}}^{2}}$, *λ*_1_ and *λ*_2_ are the eigenvalues of the Hessian matrix, *λ*_1_ < *λ*_2_. And $\text{FAH}=\sqrt{2}\mathrm{norm}\left(\tilde{H}\right)/\mathrm{norm}\left(H\right)=\sqrt{{\left(\lambda_{1}-\lambda_{2}\right)}^{2}/\left({\lambda_{1}}^{2}+{\lambda_{2}}^{2}\right)}$, which is used to describe the fractional anisotropy of the image. *c* is a free parameter, which is set to 0.2. When |*λ*_1_| ≈ 0 and |*λ*_1_|<<|*λ*_2_|, the value of FAH increases gradually from 0 to 1, the anisotropy of the Hessian matrix increases, and the target contour changes gradually from circular to linear. If FAH = 1, this point has the highest probability of belonging to a blood vessel.

**Figure 3:**
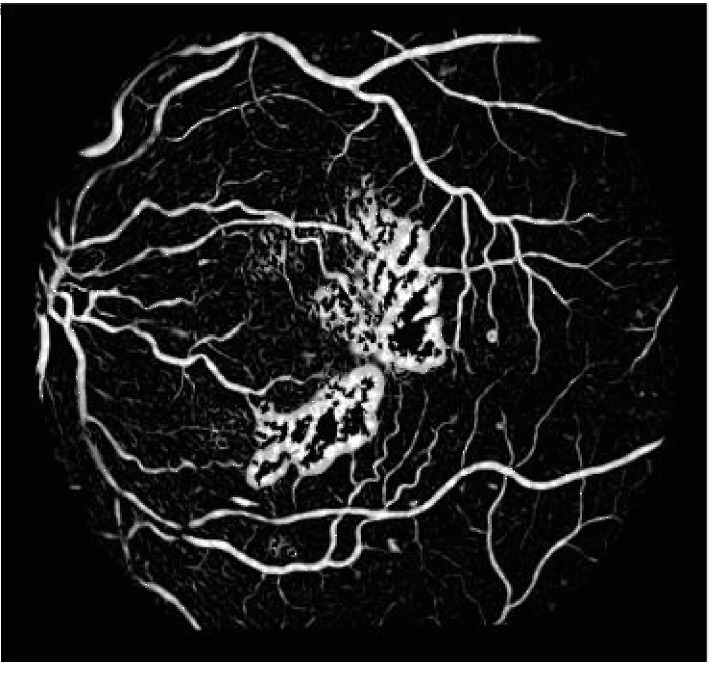
The enhanced result of the input image.

Because the size of blood vessels varies, the similarity function *ν*(*σ*) containing different values of *σ* is still used to multiscale enhance the image, and the maximum response *ν*_max_(*σ*) in this series of results is used as the final tubular feature value. The highest response provided by the filter at different scales is considered as the final output of the vesselness enhancement.  Figure 3 shows the enhanced result of the input image.

### 2.3. Vessel Segmentation

In [26], random walk algorithm was first proposed for image segmentation. Labeled seeds in this step are planted automatically based on the location of centerlines of the vessels. And the centerlines are extracted based on the enhanced images and the normalized vector field.

#### 2.3.1. Locating the Centerlines

**Figure 4:**
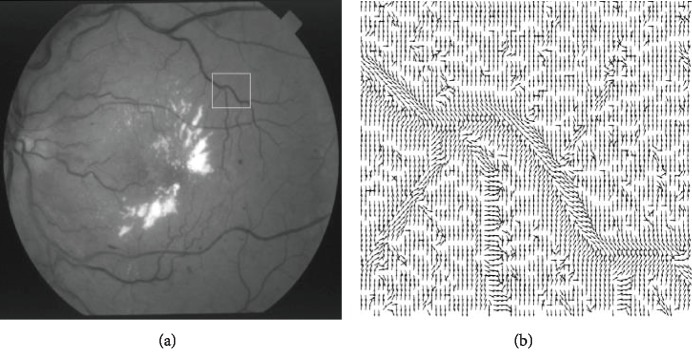
Illustration of the normalized gradient vector field in part regions of the vessels: (a) the selected region in the input image; (b) the normalized gradient vector field in the selected region.

A method proposed in [27] uses the normalized gradient vector field to locate the centerlines. If a pixel is in an expanding vector field, the divergence will be positive. The illustration of the normalized gradient vector field inside the selected local region of vessels has been shown in Figure 4. Thus, the vessels can be detected by checking whether the divergence is positive.

In order to detect the centerlines of the blood vessels in the retinal image, a horizontal and vertical edge detector is applied to *g*, the smoothed image by using the Gaussian filter, to obtain the gradient vector field *F*, where

(3) $$\begin{array}{c} F=\frac{\partial g\left(x,y,\sigma^{2}\right)}{\partial x}i+\frac{\partial g\left(x,y,\sigma^{2}\right)}{\partial y}j. \end{array}$$

Then, a set of rotation matrixes with different angles *θ* are applied to the gradient vector field, the divergence value of which can be computed as follows:

(4) $$\begin{array}{c} \text{DF}=\frac{\partial^{2}g\left(x,y,\sigma^{2}\right)}{\partial x^{2}}\cos\theta +\frac{\partial^{2}g\left(x,y,\sigma^{2}\right)}{\partial y^{2}}\sin\theta . \end{array}$$

For the normalized gradient vector field, which is calculated as NF = *F*/|*F*|, the divergence value can be calculated by the same procedure above and denoted as NG(*θ*, *σ*^2^). For each pair (*θ*, *σ*^2^), a pixel is considered in the candidate of centerlines if it satisfies NG(*θ*, *σ*^2^) > *φ* (*φ* is a user-defined parameter), and those eight-connected regions are discarded if they are less than a certain pixels *δ*.

**Figure 5:**
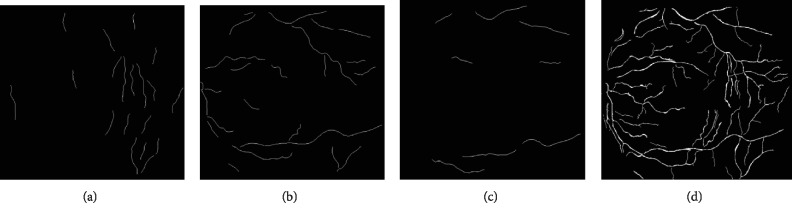
Detected candidate centerlines: the extracted centerlines with (a) *θ* = (1/8)∗*π*, *σ* = 1.4; (b) *θ* = (3/8)∗*π*, *σ* = 2.4; (c) *θ* = (4/8)∗*π*, *σ* = 4.6; (d) the combination of all the detected candidate centerlines.

In order to retain more vascular details, *φ* is set to a lower value of 0.45. Since the larger the value of *δ*, the more artifacts are removed, *δ* is set to 50. According to these parameter settings, the final candidate centerlines, *C*_can_, are obtained by combining all the 200 centerline results for each pair (*θ*, *σ*^2^).  Figure 5 shows the final candidate centerlines and with different parameters. *C*_can_ contains most of the centerlines of the vessels, with a good performance on the smoothness and connectivity. However, this algorithm cannot separate the centerlines from its neighbor centerlines if they are too close to each other.

Based on the observation above, we extract the skeleton of the vessel as the correction to the candidate centerlines as follows:

**Step 1 .**

A sum of bottom-hat operators with different orientations and scales on the green channel of the retinal images is used to enhance the vessels. Equation (5) represents the bottom-hat operator:

(5) $$\begin{array}{c} I_{\text{bot}}=\left(\upsilon •\text{Se}\right)-\upsilon , \end{array}$$

where *υ* is the input image, Se are linear structuring elements for closing (•). Because the direction of blood vessel distribution is different and the diameter of the tube is different, this paper uses multiscale and multidirectional linear structural elements. The scale ranges from 2 to 12 pixels, and the step size is 2 pixels. The direction is in the range from 0 to *π*, and the step size is *π*/8. We transformed *I*_bot_ into binary image *I*_binary_ by the OTSU method [28]. And then, morphological thinning is employed to *I*_binary_ to obtain the skeleton of the vascular tree, *C*_skel_.

**Step 2 .**

The intersection of *C*_skel_ and *C*_can_ is first calculated as *C*_inter_. And *C*_skel_ and *C*_can_ are then separated into four-connected regions. Only those regions that have more than 40% intersection with *C*_inter_ are saved, and the isolate short branches are removed by the length filter [13] threshold of which is denoted as *L*.

**Figure 6:**
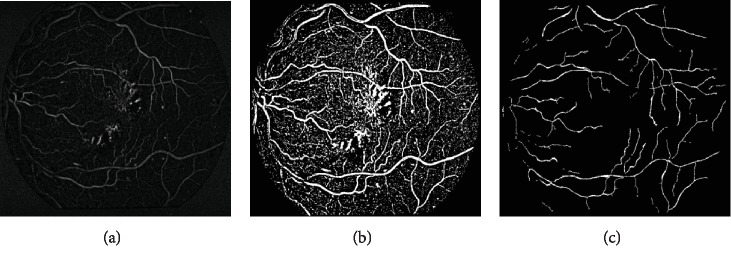
Centerline generation of the input image: (a) the result of bottom-hat transforming; (b) binary image transformed by the OTSU method; (c) the extracted centerlines.

The pseudocode of the correction strategy of centerlines is given in Pseudocode 1:

The results of bottom-hat transforming and binary image transforming are shown in Figures 6(a) and 6(b). The final true centerlines are shown in Figure 6(c).

#### 2.3.2. Random Walker Segmentation

In this step, the enhanced image obtained from the Hessian-based tubular filtering is used as the guide image for the random walker. The input image is considered a weighted graph with nodes and edges. And an 8-connected lattice is employed as the neighborhood structure. The weight of each edge expresses the similarity of intensity between the adjacent pixels *i* and *j*. Here, *w*_*ij*_ is computed as follows:

(6) $$\begin{array}{c} w_{ij}=e^{-\beta \sqrt{{\left(\upsilon_{i}-\upsilon_{j}\right)}^{2}}}, \end{array}$$

where *υ* indicates the highest response of the Hessian-based tubular filtered image at the pixel. *β* is a free parameter, which is usually set as 90.

The solution to the minimization of the following energy function is considered to be the desired random walker probabilities:

(7) $$\begin{array}{c} E\left(u\right)=\frac{1}{2}\overset{\left|L\right|}{\underset{i=1}{\sum }}\overset{\left|L\right|}{\underset{j=1}{\sum }}w_{ij}{\left(u_{i}-u_{j}\right)}^{2}, \end{array}$$

where *u*_*i*_ = 1 if pixel *i* is in a vessel and *u*_*i*_ = 0 if *i* is a background pixel. *L* is the Laplacian matrix of the edge-to-edge combination [26]. The two nodes on the edge *e*_*ij*_ are pixels *i* and *j*, respectively.

**Figure 7:**
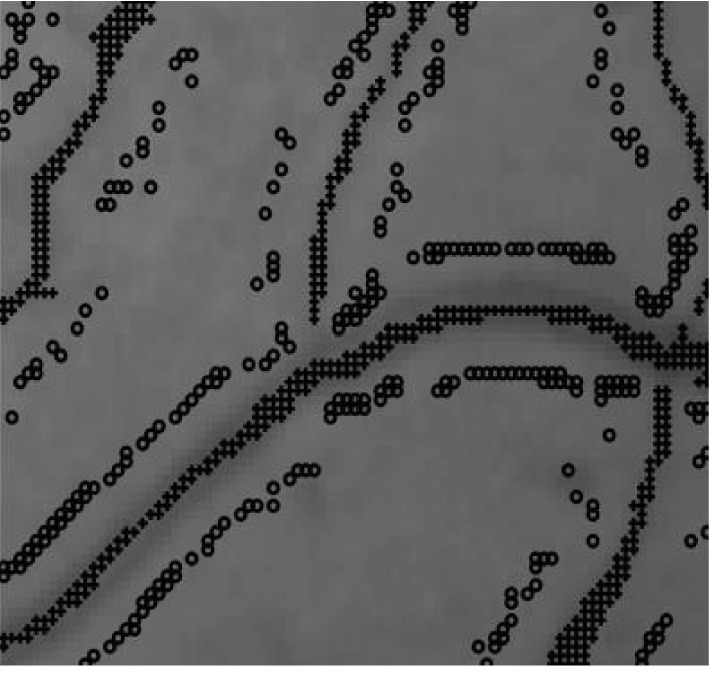
The seeds group in part of image.

The labeled pixels, foreground seeds and back ground seeds, are generated automatically based on the centerlines and the orientation information in our method. The illustration of the seeds location in a certain selected region is shown in Figure 7. The labeled pixels marked with the symbol of “+” in the darker areas are the foreground seeds, otherwise the background seeds marked with “∘.”.

Each pixel on the centerlines of a vessel is considered a foreground seed. The background seeds are planted as the following two steps:

**Step 3 .**

Areas which contain more details in the retinal image are selected. As the radius of vessels in the retinal image ranges from *R*_min_ to *R*_max_ pixels, we consider the vessels as thinner vessels, the width of which are smaller than the middle value, *R*_mid_ pixels, otherwise, larger vessels. Firstly, the centerlines are dilated by the structuring element with a radius of *R*_mid_ pixels. Secondly, for each pixel on the centerlines, if the number of the eight-connected regions of the dilated result is more than 1 in the neighborhood window with the radius of 2∗*R*_mid_ pixels, we consider the pixel is in the dense area, which contains more details. Otherwise, it is in the sparse area.

**Step 4 .**

For each foreground seed, the candidate background seeds are located several pixels away from it in the vertical direction, the distance of which is one pixel larger than the maximum radius of all the vessels in dense and sparse areas, respectively. The corresponding eigenvectors of *λ*_1_ and *λ*_2_ can be used to denote the horizontal and the vertical orientation of a vessel, respectively.

**Figure 8:**
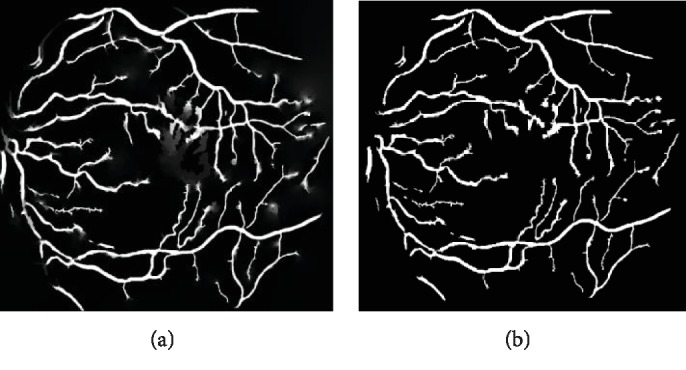
Segmentation of random walks: (a) the probability image; (b) the final segmentation results.

The pseudocode of the location strategy of background seeds is given in Pseudocode 2:

Since the width of the blood vessel ranges from 2 to 12 pixels, the *R*_min_ value is 2, and the *R*_max_ value is 12. The probability image associated with the random walker algorithm is shown in Figure 8(a). The maximum of the probabilities of the random walker is considered the final result of the segmentation, as shown in Figure 8(b).

## 3. Results and Discussion

In this work, experiments were implemented on the publicly available retinal image database, STARE. The blood vessel segmentation work includes 20 hand-labeled images provided by two experts in the database, 10 of which are pathological. We use MATLAB to implement our method on a computer with 3.2 GHz CPU and 4.00 GB RAM.

The parameter setting of the proposed algorithm in this paper is given as follows: in Equation (3), the smooth parameter *σ* = 0.2∗*m* (*m* = 1, 2, ⋯25) and the rotation parameter *θ* = *n*∗(*π*/8) (*n* = 0, 1, 2, ⋯8). The thresholding parameter *φ* = 0.45, *δ* = 50. And the maximum and the minimum values of the width of all the vessels, *R*_min_ and *R*_max_, are considered 2 and 12, respectively [29]. And the linear structuring elements used in Equation (5) are 1 pixel wide, the length of which ranges from 2 to 12 pixels with interval 2, approximately the range of the diameter of the vessels in retinal images. And we also rotated the operator at every *π*/8.

### 3.1. Qualitative Verification Compared by the Manual Results

**Figure 9:**
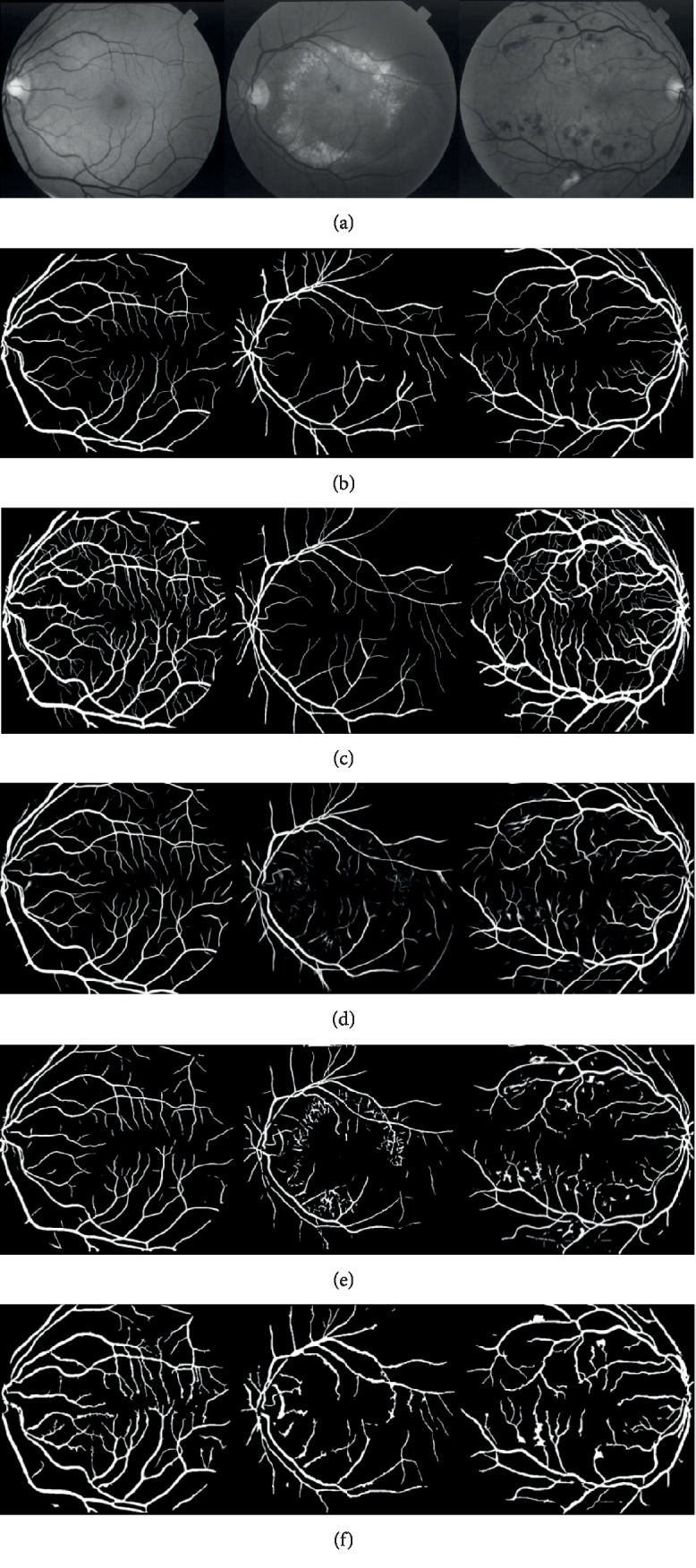
Comparison of segmentation results: (a) green channel of original images; (b) the hand-labeled ground truth segmentations labeled by Hoover; (c) the hand-labeled ground truth segmentations labeled by Valentina Kouznetsova; (d) results of the u-net [19] method; (e) results of the FC-CRF [18] method; (f) results of our method.

We have compared the results of our algorithm with several other existing approaches [2, 8, 18, 19, 30–32]. And the performance of our proposed method was tested in the region of interest (ROI) determined by the FOV mask of the retina. Since the results provided by the first expert, Hoover, are usually considered ground truth, the input images and the comparison of the segmentation between the manual work and the proposed method are given in Figure 9.

It can be seen that the main network of the vessels has been segmented in the images. And many details of the branches have also been detected.

Filters based on the eigenvalues of Hessian matrix, a square matrix of second-order partial derivatives of a scalar field, are widely applied as an efficient step in enhancing vessel structures. The random walk algorithm provides a unique and quality solution, which is robust to weak object boundaries. The labeled pixels, foreground and background seeds, are automatically planted based on the centerlines by using the information of local morphological properties, which are used as the prior knowledge in the random walk segmentation. In the proposed method, in order to improve the accuracy of the path chosen by the random walker, the vessels are also enhanced by using the multiscale tubular filters based on the Hessian matrix.

However, due to the low contrast between the vessels and the background tissue objects, methods only based on the vesselness filters tend to classify all the tubular tissues as vessels and thus would achieve high sensitivity but low specificity. And the pathological areas in the fundus images make the work of blood vessel segmentation more difficult. In our method, the divergence value of normalized gradient field is used as the threshold to help reduce the misclassification of the nonvessel objects and increase the sensitivity of the segmentation.

**Figure 10:**
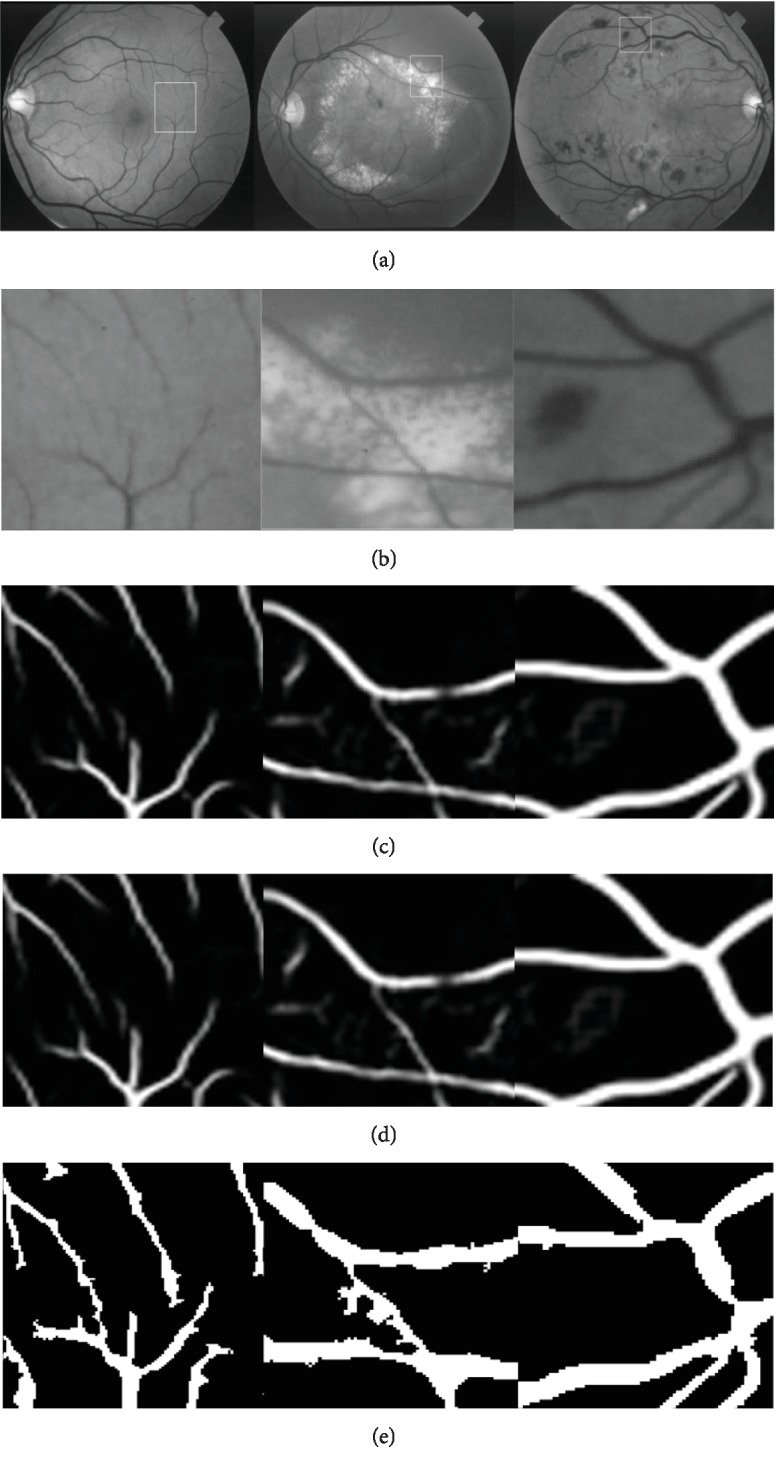
Comparison of segmentation results on some selective regions in the pathological region: (a) the green channel of the original images; (b) the centerlines extracted by the proposed method in selected regions; (c) segmentation of the u-net method; (d) segmentation of the FC-CRF method; (e) segmentation of the propose method.

As shown in Figure 10, the centerline extraction can achieve good performance in locating the vessels in both normal and pathological areas. Details of the vessels are detected in the normal areas in the first column, and the pathological tissues are not misclassified as the vessels in the second and the third columns.

Compared with other methods, the method based on the normalized gradient vector field is more obvious in the main part than the other methods and has better performance. According to the difference of the appearance between the vessel pixels and the pathological tissues in the normal gradient vector field, pixels which appear brighter than the neighbors are regarded as nonvessel pixels, which thus have good performance in specificity but not in sensitivity.

Centerlines are extracted based on the combination of the enhancing filters based on the Hessian matrix and the normalized gradient vector field, which are used to locate the vessels in the image. The candidate centerlines are obtained by considering the divergence of the normalized gradient vector field as the threshold, which can help to avoid misclassifying the nonvessel tissues. The skeletons of the tubular objects are extracted by the OTSU method, which are used as the correction and supplement to the candidate centerlines.

### 3.2. Quantitative Verification

In the retinal images, pixels correctly segmented as vessels or nonvessels are marked as true positives (TP) or true negatives (TN), respectively. Otherwise, they are marked as false negatives (FN) or false positives (FP), respectively.

In order to evaluate the performance of our proposed method, we computed the average value of accuracy (Acc), specificity (Sp), and sensitivity (Se) of the 20 test images, which are used as performance measures [2]. These metrics are defined as follows:

(8) $$\begin{array}{c} \text{Se}=\frac{\text{TP}}{\text{TP}+\text{FN}}\;\text{Sp}=\frac{\text{TN}}{\text{TN}+\text{FP}}\;\text{Acc}=\frac{\text{TP}+\text{TN}}{\text{TP}+\text{FN}+\text{TN}+\text{FP}}. \end{array}$$

In Table 1, we can see that the sensitivity of the proposed method is much higher than the other compared methods on the premise of almost equal specificity and accuracy, which means the ability of our algorithm to detect the vessel pixels is much better.

## 4. Conclusion

The segmentation work of retinal blood vessels is of great significance in the diagnoses of diseases by ophthalmologists. In our paper, based on the complex physical appearance and structure of the vessels in normal and pathological retinal images, we extract centerlines of vessels by using the combination of the multiscale enhancing filter based on Hessian matrix and the divergence value of the normalized gradient vector field, the local information of which are used to locate the labeled seeds in the random walks for segmentation. In our experiment, the results of the proposed method can achieve better performance in detecting the true vessels in both normal and pathological areas.

However, there is still room for further improvement in our method. Using the proposed segmentation method to process a fundus image with a size of 605∗700 pixels, the average time is nearly 90 seconds, so the real-time performance of the algorithm needs to be further improved. Only the ratio of the intersection between the candidate centerlines and the skeletons is considered, which may cause the loss of some details. And the distance between each pair of the foreground and background seeds is measured by parameters. If more effective approaches to implement different features of retinal blood vessels are considered in the algorithm, the performance of the final segmentation results can be better. In the future, we will combine the machine learning method to locate the blood vessel centerline and seed points, to reduce the setting of human parameters, and to improve the robustness of the algorithm.

## Figures and Tables

**Pseudocode 1:**
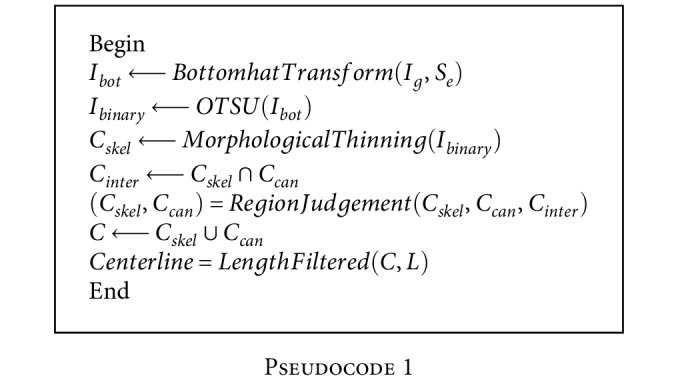
Pseudocode 1

**Pseudocode 2:**
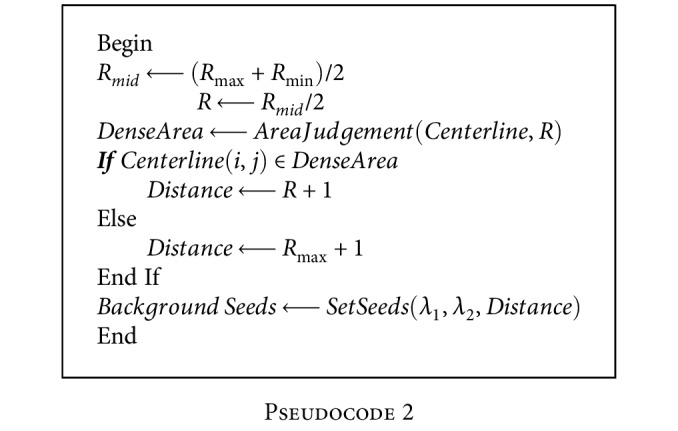
Pseudocode 2

**Table 1:** Comparative performance of proposed method with other existing methods on the STARE database.

Method	Year	Sensitivity	Specificity	Accuracy
2nd human observer	Not available	0.8952	0.9385	0.9349
Hoover et al. [8]	2000	0.6734	0.9568	0.9267
Jiang and Mojon [33]	2003	Not available	Not available	0.9009
Martinez-Perez et al. [31]	2007	0.7506	0.9569	0.9410
Al-Rawi et al. [29]	2007	Not available	Not available	0.9090
Palomera-Pérez et al. [32]	2010	0.769	0.944	0.926
Budai et al. [30]	2013	0.5800	0.9820	0.9370
Chakraborti et al. [2]	2014	0.6786	0.9586	0.9379
Soomro et al. [20]	2017	0.7480	0.9220	0.9480
Abdallah et al. [17]	2018	0.6801	0.9711	0.9388
Leopold et al. [21]	2019	0.6433	0.9472	0.9045
Proposed method	2019	0.7581	0.9550	0.9401

## Data Availability

The data used to support the findings of this study are available from the corresponding author upon request.
